# Optimization of Microwave-Assisted Extraction Conditions for Five Major Bioactive Compounds from Flos Sophorae Immaturus (Cultivars of *Sophora japonica* L.) Using Response Surface Methodology

**DOI:** 10.3390/molecules21030296

**Published:** 2016-03-02

**Authors:** Jin-Liang Liu, Long-Yun Li, Guang-Hua He

**Affiliations:** 1 Institute of Material Medical Planting, Chongqing Academy of Chinese Materia Medica (Chongqing Engineering Research Center for Fine Variety Breeding Techniques of Chinese Materia Medica, Chongqing Key Laboratory of Chinese Medicine Resources), Chongqing Sub-Center of National Resource Center for Chinese Materia Medica, China Academy of Chinese Medical Science, Chongqing 400065, China; liujinliang0827@163.com; 2 College of Agronomy and Biotechnology, Southwest University, Chongqing 400715, China

**Keywords:** Flos sophorae immaturus, response surface methodology, simultaneous extraction, microwave-assisted extraction, UHPLC-ESI-Q-TOF MS/MS, simultaneous determination

## Abstract

Microwave-assisted extraction was applied to extract rutin; quercetin; genistein; kaempferol; and isorhamnetin from Flos Sophorae Immaturus. Six independent variables; namely; solvent type; particle size; extraction frequency; liquid-to-solid ratio; microwave power; and extraction time were examined. Response surface methodology using a central composite design was employed to optimize experimental conditions (liquid-to-solid ratio; microwave power; and extraction time) based on the results of single factor tests to extract the five major components in Flos Sophorae Immaturus. Experimental data were fitted to a second-order polynomial equation using multiple regression analysis. Data were also analyzed using appropriate statistical methods. Optimal extraction conditions were as follows: extraction solvent; 100% methanol; particle size; 100 mesh; extraction frequency; 1; liquid-to-solid ratio; 50:1; microwave power; 287 W; and extraction time; 80 s. A rapid and sensitive ultra-high performance liquid chromatography method coupled with electrospray ionization quadrupole time-of-flight tandem mass spectrometry (EIS-Q-TOF MS/MS) was developed and validated for the simultaneous determination of rutin; quercetin; genistein; kaempferol; and isorhamnetin in Flos Sophorae Immaturus. Chromatographic separation was accomplished on a Kinetex C_18_ column (100 mm × 2.1 mm; 2.6 μm) at 40 °C within 5 min. The mobile phase consisted of 0.1% aqueous formic acid and acetonitrile (71:29; *v*/*v*). Isocratic elution was carried out at a flow rate of 0.35 mL/min. The constituents of Flos Sophorae Immaturus were simultaneously identified by EIS-Q-TOF MS/MS in multiple reaction monitoring mode. During quantitative analysis; all of the calibration curves showed good linear relationships (*R*^2^ > 0.999) within the tested ranges; and mean recoveries ranged from 96.0216% to 101.0601%. The precision determined through intra- and inter-day studies showed an RSD% of <2.833%. These results demonstrate that the developed method is accurate and effective and could be readily utilized for the comprehensive quality control of Flos Sophorae Immaturus.

## 1. Introduction

*Sophora japonica* L. is used in Traditional Chinese Materia Medica (TCMM), and was first officially listed in the Chinese Pharmacopoeia in 1963 [1,2,3,4,5,6,7,8,9]. The dried flower bud of *S. japonica* L. is generally called Huaimi (Flos Sophorae Immaturus, FSI) in China. In the south of China more than 13,000 hectares of this species have been artificially planted. Mainly cultivated in Chongqing City, and Quanzhou County, Guilin City, Guangxi Autonomous Region in China, and twice in 2009 and 2015 successively through the examination and approval of varieties by the forestry administration committee Chongqing city, China. The main constituents of *S. japonica* L. include rutin, quercetin, genistein, kaempferol, and isorhamnetin, among others [10,11,12,13,14,15]. FSI is used as a hemostatic agent to treat hemorrhoids and hematemesis [15,16,17,18].

Extraction of herbs for TCMM is a good technique for process engineers in production development and for product quality evaluation in pharmaceutical industry. However, simultaneous extraction of the five aforementioned contents from FSI and Sophora Flower (SF) has not been reported. Various novel techniques have recently been developed to extract one, two, or three constituents from FSI and SF [8,19,20,21,22,23,24,25,26,27,28,29,30,31,32,33,34,35,36,37,38,39,40,41,42,43,44,45,46,47,48,49,50,51,52,53,54,55,56,57,58,59,60,61]. These methods include decoction [49], percolation [22,28], reflux [25,44,46,53], Soxhlet [27,44,60], ultrasonic-assisted extraction [8,20,21,23,25,26,29,30,38,42,50,51,52,56,57,58,59,61], microwave-assisted extraction (MAE) [24,25,32,33,34,35,36,45,47,55], infrared-assisted extraction [43], supercritical fluid CO_2_ extraction [41], basic method [39,48], and enzymatic method [37] (Table 1). However, despite their advantages, these extraction methods require long extraction times [8,19,20,21,22,23,25,30,37,39,40,41,44,46,48,50,51,52,53,55,56,57,58], have low efficiency [1,19,20,22,25,26,28,39,41,44,45,47,50,60], and/or are expensive [8,21,22,23,27,37,58]. Thus, developing a reliable, economical, efficient, and ecologically sensitive technique for extracting the constituents of FSI is necessary.

MAE is widely used to extract active constituents from various kinds of plant materials because of its enhanced extraction efficiency compared with other traditional extraction methods [62,63,64,65,66]. The MAE system rapidly generates heat and can facilitate the penetration of solvent into raw plant material and intracellular material to improve constituent transfer, reducing extraction time and improving extraction rate [62,63]. The efficiency of MAE depends on extraction time, liquid-to-solid ratio, extraction power, and type of extraction solvent [62,63,64,67]. In this study, we investigated the MAE of major constituents from FSI and SF. Response surface methodology (RSM) was used to improve the extraction yield of constituents by systematically analyzing the effects of extraction parameters on yields. RSM is a collection of statistical, as well as mathematical, techniques and effective for responses that are influenced by various factors and their interactions [68,69].

The active compounds of herbs vary according to several factors, including variety, geographic area, nutritional status, harvest time, manufacturing process, and even storage method [70]. Variations in these factors could result in significant differences in pharmacological activity. Accurate, analytically obtained qualitative and quantitative data are used to evaluate the efficacy and safety of TCMM [71,72,73,74,75,76,77,78,79]. Therefore, developing a reliable and accurate quality control method for TCMM is necessary. Previous, analytical methods developed for determining the quality of a few components in FSI include HPLC-UV [20,80,81,82], HPLC-DAD [21,23,59], HPLC-DAD-ESI-MS/MS [83], and capillary electrophoresis [17,84,85]. While these reported methods contribute significantly to the current knowledge of FSI compounds, several drawbacks, such as identification of too few constituents, long analysis times, and high solvent consumption, limit their practical application. Thus, a new analytical method must be developed to qualitatively and quantitatively determine multiple active constituents in FSI.

Ultra-high performance liquid chromatography coupled with electrospray ionization quadrupole time-of-flight tandem mass spectrometry (UHPLC-ESI-Q-TOF MS/MS) is a powerful approach that enables simultaneous determination of multiple components [86,87,88,89,90,91,92]. The chemical structures of FSI constituents could readily be defined by using fragmentation rules, characteristic fragmentation and quasi-molecular ions summarized in the literature, and comparison of retention times and parent and product ions with those of standards.

This study aimed to investigate the significant variables (methanol and ethanol concentrations, particle size, extraction frequency, liquid-to-solid ratio, microwave power, and extraction time) prior to RSM to optimize variables for FSI extraction and developed a simple and accurate UHPLC and LC-ESI-Q-TOF MS/MS method for simultaneously determining five components of FSI.

## 2. Results and Discussion

### 2.1 Analysis of Single Factor Test Results

#### 2.1.1. Effect of Solvent Type on Extraction

**Figure 1:**
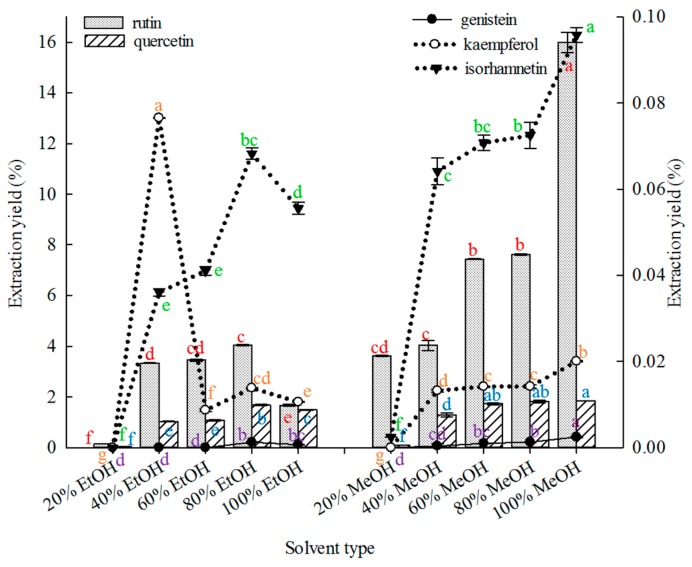
Effects of solvent type on the extraction yields of the five constituents (the bar and lines graphs as referenced to the left and right axes, respectively; different letters stand for significant difference at 5% level).

Solvent selection is important in the extraction of compounds from botanical materials [69]. Figure 1 shows that under similar extraction conditions. 100% MeOH was superior to other solvents in extracting rutin, genistein and isorhamnetin from FSI. Both 80% MeOH and 100% MeOH were superior to the other solvents in extracting quercetin, but without significant differences in their extraction yields (*p* ≤ 0.01). The 40% EtOH solvent was superior to the other solvents in extracting kaempferol.

Thus, 100% methanol was selected as the extraction solvent to extract the five major constituents using MAE in subsequent experiments. Previous studies reported the use of different ethanol and methanol concentrations mainly for extracting rutin or quercetin from SFI [19,20,21,22,23,25,26,27,28,32,34,35,40,43,45,46,47,58,59,60]. Water [31,33,36,42], alkaline solution [29,30,38,48], cellulase [37], ether [41], and sodium hydroxide [49] have also been reported as extraction solvents for extracting ingredients from SFI. However, ether and alkaline solutions are toxic, water cannot dissolve flavonoid components, and cellulase is expensive. Although the methanol and ethanol concentrations used were somewhat variable, methanol was selected as the best extraction solvent among the extraction solvents listed in Section 3.2.1.

#### 2.1.2. Effect of Particle Size on Extraction

**Figure 2:**
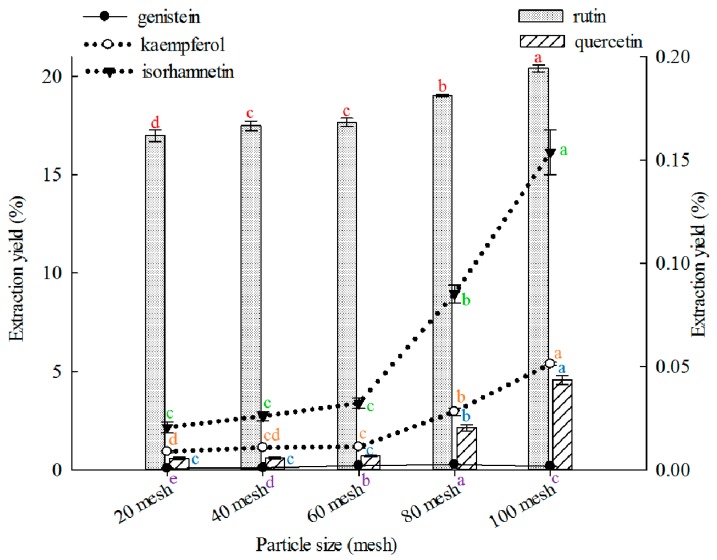
Effects of particle size on the extraction yields of the five constituents (the bar and lines graphs are referenced to the left and right axes, respectively; different letters stand for significant difference at 5% level).

The appropriate particle size is fundamental to obtain optimal extraction, and varied particle sizes can significantly affect extraction yields [69]. In this study, 100 mesh showed better results than other particle sizes in extracting rutin, quercetin, kaempferol, and isorhamnetin (Figure 2), and 80 mesh was better than other particle sizes in extracting genistein. Effects of particle size on extraction of rutin, quercetin, genistein, kaempferol, or isorhamnetin from FSI are rarely reported.

#### 2.1.3. Effect of Frequency on Extraction

**Figure 3:**
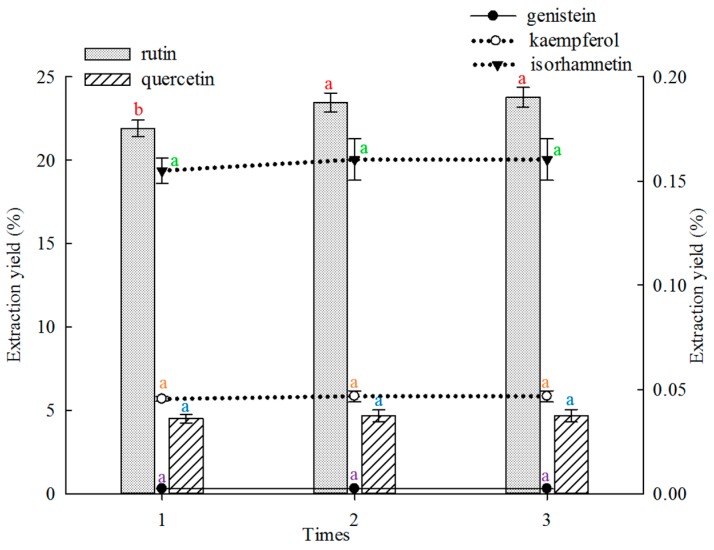
Effects of times on extraction yields of the five constituents (the bar and lines graphs are referenced to the left and right axes, respectively; different letters stand for significant difference at 5% level).

The effect of extraction frequency on the extraction yield of the five main constituents from FSI was investigated, and the results are shown in Figure 3. In this study, twice and thrice extraction showed better results in extracting rutin than single extraction. The twice extraction and thrice extraction did not differ significantly. The extraction yields of quercetin, genistein, kaempferol and isorhamnetin did not differ significantly when the extraction was performed once to thrice.

#### 2.1.4. Effect of Liquid-to-Solid Ratio on Extraction

**Figure 4:**
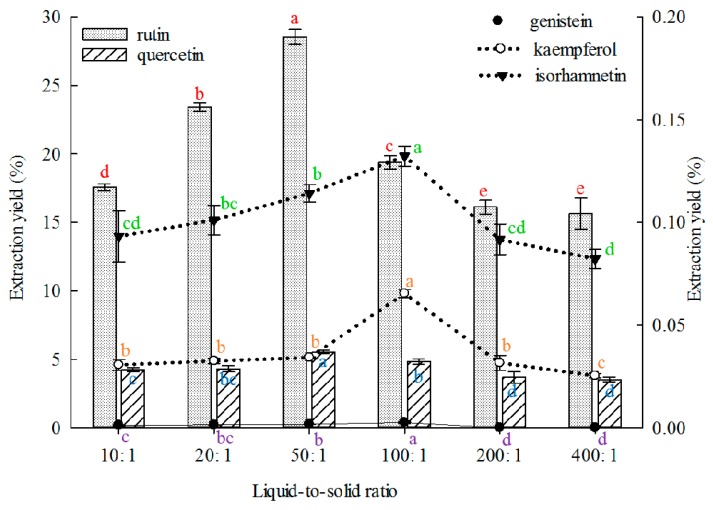
Effects of liquid-to-solid ratio on the extraction yields of the five constituents (the bar and lines graphs are referenced to the left and right axes, respectively; different letters stand for significant difference at 5% level).

The solvent volume must be sufficient to ensure complete immersion of materials for efficient extraction. Extraction solvent deficiency can lead to incomplete extraction of ingredients, but redundant solvent may also lead to lower extraction yields and solvent waste [69]. Therefore, the liquid-to-solid ratio must be appropriate. The effect of liquid-to-solid ratio on the extraction yield of the five constituents was investigated, and the results are shown in Figure 4. The 50:1 (*v*/*m*) proportion showed better results than other liquid-to-solid ratios in extracting rutin and quercetin. The 100:1 proportions were better than other liquid-to-solid ratios in extracting genistein, kaempferol and isorhamnetin. The maximum and minimum liquid-to-solid ratios of 500:1 and 4:1, respectively, have been reported for the extraction of rutin or quercetin from FSI, but with low extraction yields [21,47].

#### 2.1.5. Effect of Microwave Power on Extraction

**Figure 5:**
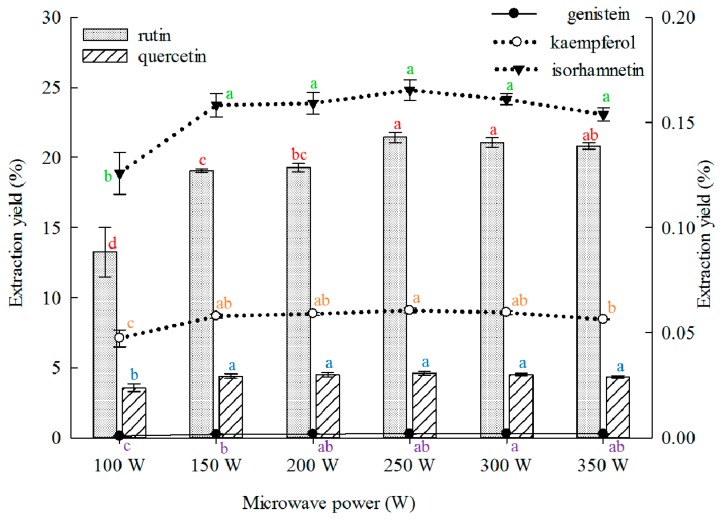
Effect of microwave power on the extraction yields of the five constituents (the bar and lines graphs are referenced to the left and right axes, respectively; different letters stand for significant difference at 5% level).

Low microwave power reduces extraction yield. However, excessively high power results in energy wastage. Therefore, the optimal microwave power should be determined. The effect of microwave power on the extraction yield of the five main constituents from the FSI was investigated, and the results are shown in Figure 5. The extraction yields of rutin, quercetin, kaempferol, and isorhamnetin from the FSI evidently increased with increasing microwave power, but no increase was observed above 250 W. The extraction yield of genistein continued to increase with the microwave power and peaked when the power was 300 W. Subsequently, a reduction in yield was observed. Previous study on the effects of microwave power for rutin and quercetin extraction from FSI are available, but microwave power was high (350–480 W) and the extraction yield was low (rutin, 14.66%–21.97%; quercetin, 0.46%–0.61%) [31,36,45,47].

#### 2.1.6. Effect of Time on Extraction

**Figure 6:**
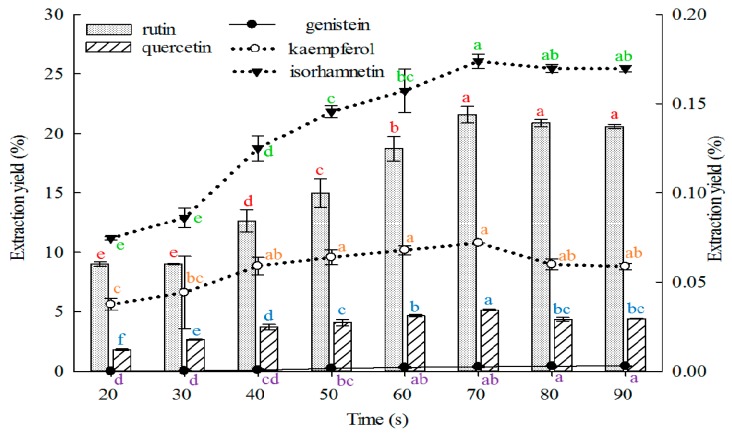
Effect of time on the extraction yields of the five constituents (the bar and lines graphs are referenced to the left and right axes, respectively; different letters stand for significant difference at 5% level).

Longer time indicates greater contact between the solvent and sample contact. This phenomenon may accelerate the absorption of solvent, soften the plant tissues, and weaken the cell wall integrity. Moreover, ingredient solubility may be enhanced, thus larger amounts of substances are distributed to the solvent. However, excessive extraction time may lead to lower process efficiency and wasted time. Therefore, the extraction time must be appropriate. The effects of extraction time on the extraction yields of the five constituents from FSI are shown in Figure 6.

The extraction yields of rutin, quercetin, kaempferol, and isorhamnetin increased as the extraction time was prolonged from 20 s to 70 s and peaked at 70 s. However, extraction yields of genistein increased as the extraction time was increased from 20 s to 80 s and peaked at 80 and 90 s, but without a statistically significant difference. Thus, extraction time is shorter than those reported in previous reports [8,19,20,21,22,23,25,30,37,39,40,41,44,46,48,50,51,52,53,55,56,57,58].

### 2.2. Model Fitting of Parameters Based on the Extraction Yields of the Five Constituents

The responses of the five extracts in each run are presented in Table 2. The regression coefficients and results from ANOVA of the second-order polynomial models (Y = A_0_ + A_1_X_1_ + A_2_X_2_ + A_3_X_3_ + A_11_X_1_^2^ + A_22_X_2_^2^ + A_33_X_3_^2^ + A_12_X_1_X_2_ + A_13_X_1_X_3_ + A_23_X_2_X_3_) for five extracts are summarized in Table 3. Regression parameters of the surface response analysis of the linear and quadratic models, and their corresponding interaction terms showed significant differences (*p* ≤ 0.0001, *p* ≤ 0.01 or *p* ≤ 0.05). The fitness of the model was evaluated through the lack of fit test (*p* < 0.05), which indicates the adequacy of the model to predict the variation accurately [93]. The models were used to construct three-dimensional response surface plots to predict the relationship between independent and dependent variables.

#### 2.2.1. Effect of Process Variables on the Extraction Yield of Rutin

The experimental data were examined through regression analysis, and the coefficients of the model were evaluated for significance. Liquid-to-solid ratio (*X*_1_), microwave power (*X*_2_), and time (*X*_3_) significantly affected the extraction yield of rutin (*Y*_1_, Table 3), with corresponding contribution rates of 2.88, 2.90, and 2.92. These results indicate that microwave power and extraction time exhibited the greatest effect on the extraction yield of rutin.

**Figure 7:**
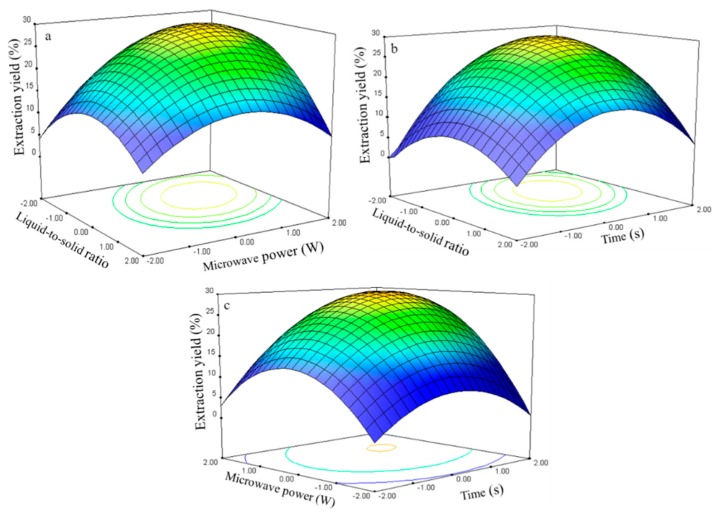
Response surface plots for the effects of: (**a**) liquid-to-solid ratio/power (**b**) liquid-to-solid/time (**c**) power/time on the extraction yield of rutin.

The three-dimensional response surface plots in Figure 7 illustrate the relationship between the extraction yield of rutin and experimental variables. Figure 7a illustrates the interaction effect of liquid-to-solid ratio and power on the extraction yield of rutin when the time was set to its 0 level (70 s). Rutin yields gradually increased with liquid-to-solid ratio and microwave power and peaked at approximately 50:1 to 75:1 and 250 W to 300 W. However, extraction yield of rutin began to decrease after further increase in these parameters.

The interaction effects between the liquid-to-solid ratio and the time on the rutin extraction yield when the power was set to its 0 level (250 W) are presented in Figure 7b. The rutin yield increased and peaked at 50:1 to 100:1 from 75 s to 80 s.

The interaction effects of power and time at 75:1 (0 level) on the rutin extraction yield are presented in Figure 7c. Strong interaction was observed when the power was set from 275 W to 300 W and the time ranged from 75 s to 80 s, which contributed to the increased extraction yield.

The rutin regression model for the statistical frequency method of analysis with 95% confidence interval was obtained (*X*_1_: –1.47 to –0.58, *X*_2_: 0.72 to 1.26, and *X*_3_: 0.64 to 1.18) when the extraction yield was >26.80% (*n* = 21). Therefore, the optimal conditions were 46.33–60.60, 286.05–312.85W, and 76.35–81.83 s.

#### 2.2.2. Effect of Process Variables on the Extraction Yield of Quercetin

**Figure 8:**
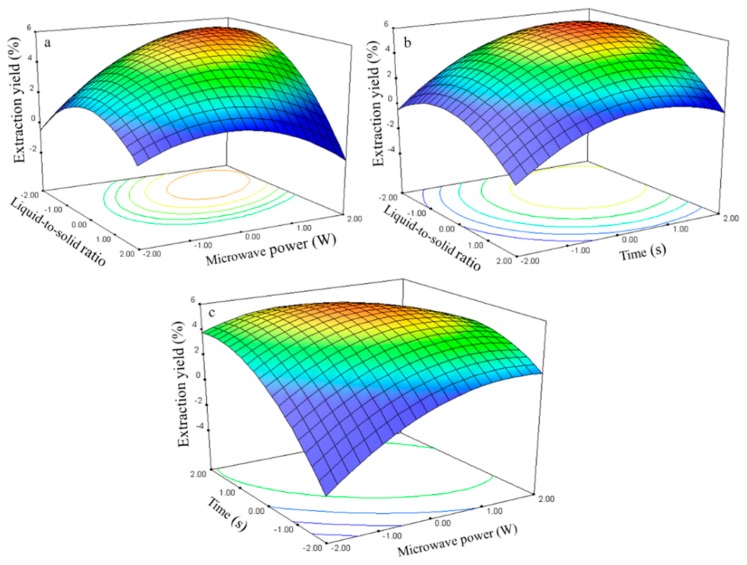
Response surface plots for the effects of: (**a**) liquid-to-solid ratio/power (**b**) liquid-to-solid/time (**c**) power/time on the extraction yield of quercetin.

The quercetin extraction yield results are presented in Table 2. The regression analysis results indicate that the main extraction parameters of quercetin are the liquid-to-solid ratio (*X*_1_), microwave power (*X*_2_), and time (*X*_3_). The relationships between the quercetin extraction yield (*Y*_2_, Table 3) and the variables are shown in Figure 8. The contributions of the liquid-to-solid ratio, microwave power, and extraction time were 2.79, 2.95, and 2.80, respectively. Microwave power had the largest impact on the quercetin extraction yield.

The effect of the liquid-to-solid ratio and microwave power on the quercetin extraction yield at a constant time (0 level) is shown in Figure 8a. The quercetin extraction yield gradually increased with the ratio and power and peaked at approximately 45–55 and 260–300 W, respectively. The quercetin extraction yield began to decrease beyond 55 and 300 W. The appropriate extraction time (75–80 s) had positive effects on the extraction yield, as shown in the response surface plots for the time effect on the extraction yield (Figure 8b,c) at constant microwave power and liquid-to-solid ratio.

The quercetin regression model for the statistical frequency method of analysis with 95% confidence interval was obtained (*X*_1_: −1.11 to −0.53, *X*_2_: 0.23 to 0.95 and *X*_3_: 0.51 to 1.10) when the extraction yield was >5% (*n* = 20). Thus, the optimal conditions were 47.28–61.70, 261.35–297.30 W, and 75.10–80.63 s.

#### 2.2.3. Effect of Process Variables on the Extraction Yield of Genistein

The genistein extraction yield is presented in Table 2. The regression analysis shows that the extraction yield (*Y*_3_, Table 3) was significantly affected by the liquid-to-solid ratio (*X_1_*), microwave power (*X*_2_), and time (*X*_3_), with corresponding contribution rates of 2.88, 1.91, and 2.86, respectively. The liquid-to-solid ratio and extraction time exhibited the largest impact on the genistein extraction yield.

**Figure 9:**
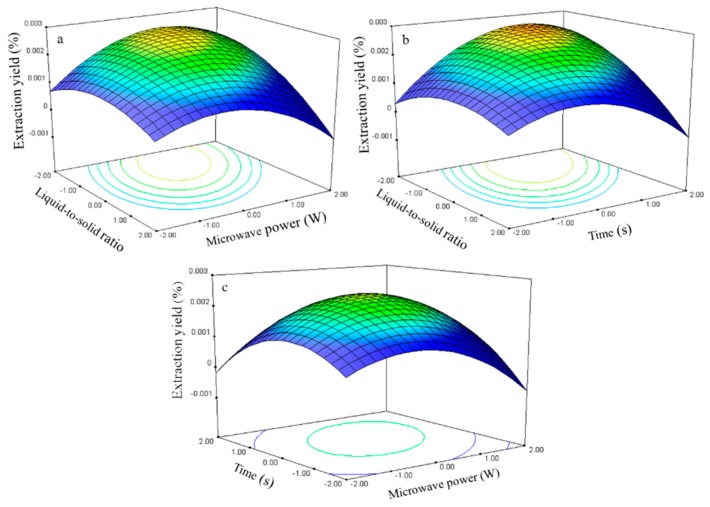
Response surface plots for the effects of: (**a**) liquid-to-solid ratio/power (**b**) liquid-to-solid/time (**c**) power/time on the extraction yield of genistein.

The relationship between the genistein extraction yield and the process variables is depicted in Figure 9. The liquid-to-solid ratio and microwave power effects on the extraction yield at 0 level fixed time are shown in Figure 9a. The genistein extraction yield gradually increased with the ratio and power and peaked at approximately 50–60 and 270–300 W. Further increases in these parameters resulted in decreased genistein extraction yield.

The response surface plots for the time effect on the extraction yield (Figure 9b,c) at constant microwave power and liquid-to-solid ratio show that an appropriate extraction time (75–80 s) had positive effects on the extraction yield.

The genistein regression model for the statistical frequency method of analysis with 95% confidence interval was obtained (*X*_1_: −1.31 to −0.79, *X*_2_: 0.30 to 0.96, and *X*_3_: 0.51 to 1.10) when the extraction yield >0.002% (*n* = 23). Thus, the optimal conditions were 42.33–55.20, 264.75–297.90 W, and 75.07–80.66 s.

#### 2.2.4. Effect of Process Variables on the Extraction Yield of Kaempferol

The kaempferol extraction yield is presented in Table 2. The regression analysis shows that the extraction yield (*Y*_4_, Table 3) was significantly affected by the liquid-to-solid ratio (*X*_1_), microwave power (*X*_2_), and time (*X*_3_), with corresponding contribution rates of 1.06, 1.93, and 2.81. The extraction time had the largest impact on the kaempferol extraction yield.

**Figure 10:**
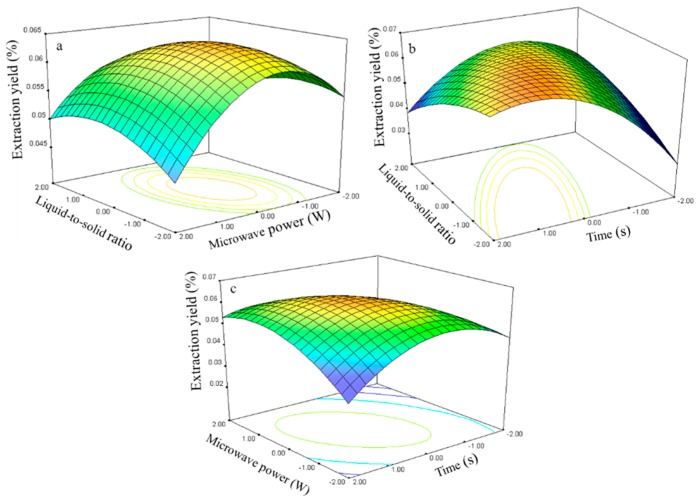
Response surface plots for the effects of: (**a**) liquid-to-solid ratio/power (**b**) liquid-to-solid/time (**c**) power/time on the extraction yield of kaempferol.

The relationship between the kaempferol extraction yield and the process variables is depicted in Figure 10. The ratio and power effects on the extraction yield at 0 level fixed time are shown in Figure 10a.

The extraction yield gradually increased with the ratio and power and peaked at approximately 50–75 and 250–300 W. Further increases in these parameters resulted in decreased kaempferol extraction yields. The response surface plots for the time effect on the extraction yield (Figure 10b,c) at constant microwave power and constant liquid-to-solid ratio show that an appropriate extraction time (75–80 s) had positive effects on the extraction yield.

The kaempferol regression model for the statistical frequency method of analysis with 95% confidence interval was obtained (*X*_1_: −1.14 to −0.17, *X*_2_: 0.11 to 0.78, and *X*_3_: 0.44 to 1.12) when the extraction yield was >0.06% (*n* = 22). Thus, the optimal conditions were 46.5–70.75, 255.45–288.30 W, and 74.35–81.18 s.

#### 2.2.5. Effect of Process Variables on the Extraction Yield of Isorhamnetin

A regression analysis was performed using the experimental data, and the model coefficients were evaluated for significance. The liquid-to-solid ratio (*X*_1_), microwave power (*X*_2_), and time (*X*_3_) significantly affected the isorhamnetin extraction yield (*Y*_5_, Table 3), with corresponding contribution rates of 2.85, 2.44, and 2.45. The liquid-to-solid ratio had the largest impact on the isorhamnetin extraction yield.

**Figure 11:**
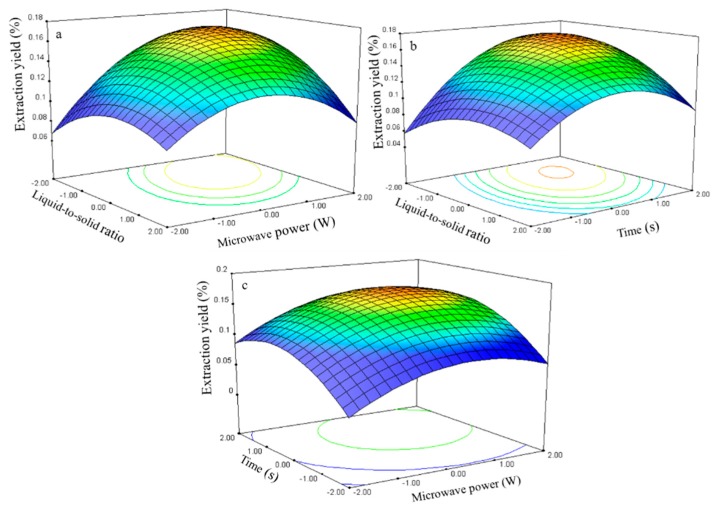
Response surface plots for the effects of (**a**) liquid-to-solid ratio/power (**b**) liquid-to-solid/time (**c**) power/time on the extraction yield of isorhamnetin.

The three-dimensional response surface plots (Figure 11) illustrate the relationship between the isorhamnetin extraction yield and experimental variables. These plots present the response as a function of two factors with another variable constant at its 0 level. Figure 11a shows the interaction effect between the ratio and power when the time was set at its 0 level (70 s) in the isorhamnetin extraction. The isorhamnetin extraction yield gradually increased with the ratio and power and peaked at approximately 40–55 and 275–300 W.

The interaction effect between liquid-to-solid ratio and time at 0 level power (250 W) on isorhamnetin extraction is presented in Figure 11b. The response surface plot shows that the extraction yield of isorhamnetin increased and reached the maximum level at 50–60 for the time interval of 75–80 s.

The interaction effect between power and time at the 0 level liquid-to-solid ratio (75) on isorhamnetin extraction yield is presented in Figure 11c. Strong interaction was observed when the power was set from 250 W to 300 W and time ranged from 70 s to 80 s, which contributed to the increase in extraction yield.

The isorhamnetin regression model for the statistical frequency method of analysis with 95% confidence interval was obtained (*X*_1_: –1.33 to –0.81, *X*_2_: 0.58 to 1.15, and *X*_3_: 0.58 to 1.15) when the extraction yield was >0.16% (*n* = 21). Thus, the optimal conditions were 41.83–54.70, 278.80–307.35 W, and 75.76–81.47 s.

### 2.3. Optimization of the Extraction Process

Table 4 indicates the optimum microwave-assisted conditions for the extraction of the five major constituents from FSI using RSM. Thirty accurately weighed samples (*i.e.*, 0.5 g samples filtered through a 100 mesh sieve) were added to 25 mL 100% MeOH and divided into six groups. A sample set was extracted under the optimum single ingredient conditions (obtained using statistical software), and the predicted results fitted well with the experimental results (Table 4).

The following optimum extraction conditions were obtained on the basis of the statistics frequency method: liquid-to-solid ratio, 50; microwave power, 287 W; and extraction time, 80 s. Six accurately weighed samples (*i.e*., 0.5 g samples filtered through a 100 mesh sieve) were added to 25 mL of extracted 100% MeOH. The optimum extraction conditions were obtained through the statistics frequency method (Table 4). The calculated extraction yields of the statistical frequency condition were compared with the actual extraction yields under optimum conditions.

The results show that the significant results obtained in the genistein extraction yields are insignificant unlike the other four constituents. Therefore, the optimum extraction conditions for the simultaneous extraction of the five ingredients can be obtained through the statistical frequency method. The optimum extraction condition for one ingredient can be obtained through the single-component method.

With the development of the “green chemistry” and “green extraction” concept during the past years, reduce energy consumption, safe, robust, and environmentally friendly extraction techniques are becoming more toward and popular [94,95]. Green extraction is based on the discovery and design of extraction processes, which will reduce energy consumption, allows use of alternative solvents and renewable natural products, and ensure a safe and high quality of extract/product [94,95]. The principles of green extraction are renewable plant resource instead of non-renewable resources, green and alternative solvents, reduce energy consumption and heat production, safe and robust to extraction processes [94]. In the present study, six factors, namely extraction solvent type, sample particle size, frequency, liquid-to-solid ratio, microwave power, and extraction time, were investigated first to optimize the extraction solvent type, sample particle size, and frequency. Additionally, the levels of the response surface experimental design factors (liquid-to-solid ratio, microwave power, and time) were determined according to the liquid-to-solid ratio, microwave power, and extraction time optimized in the single factor tests. Higher yields of the constituents were green extracted from FSI using a renewable raw material, green extraction solvent, less solvent, lower extraction power, reduce energy consumption, more economical, and simultaneously and considerably decreasing the extraction time. TCMM and natural product green extraction, according to the principle of green extraction, is a new concept to meet the future trends [94,95].

### 2.4. Optimization of UHPLC-ESI-Q-TOF MS/MS Conditions

Isocratic and gradient LC were tested to optimize the separation conditions of all constituents. Various UHPLC conditions, such as mobile phase, gradient program, column, column temperature, and flow rate were optimized systematically in a preliminary test to improve the separation efficiency of analytes over short analysis times. Two analytical columns, namely, an Acquity UPLC^®^ BEH C_18_ column (100 mm × 2.1 mm, 1.7 μm, Waters, Wexford, Ireland) and a Kinetex C_18_ column (100 mm × 2.1 mm, 2.6 μm, Phenomenex, Torrance, CA, USA), were compared. Results showed that although all of the compounds could be separated satisfactorily, chromatograms with better peak shapes and shorter analysis times were obtained with the Acquity UPLC^®^ BEH C_18_ column (100 mm × 2.1 mm, 1.7 μm). Thus, this column was selected as the analytical column in subsequent experiments.

**Figure 12:**
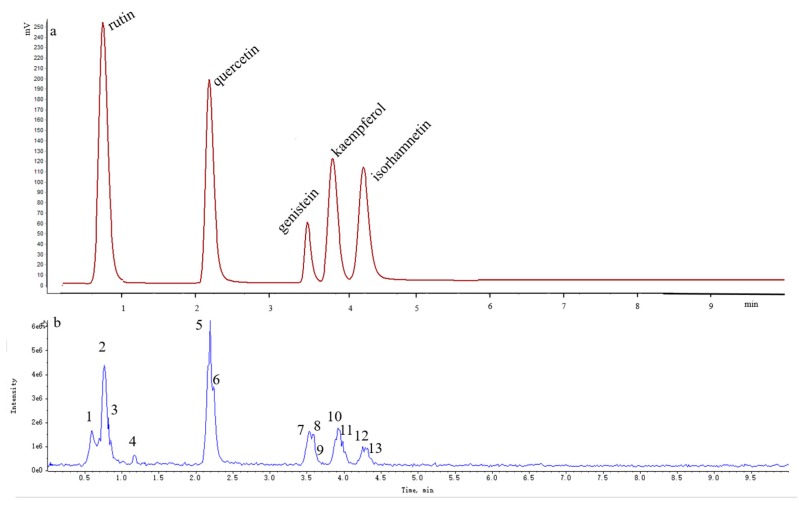
UHPLC (**a**) and LC-ESI-Q-TOF/MS TIC (**b**) chromatography of FSI.

Different mobile phases (water/acetonitrile, water/methanol, 0.1% formic acid/acetonitrile, 0.1% formic acid/methanol, 0.2% phosphate/acetonitrile, 0.2% phosphate/methanol, 0.5% acetic acid/acetonitrile, 0.5% acetic acid/methanol), flow rates (0.10, 0.12, 0.15, 0.20, 0.25, 0.30, 0.35, 0.40, and 0.45 mL/min), and column temperatures (30, 35, 40, 45, 50, 55, and 60 °C) were also examined and compared. Considering the results obtained, we selected the isocratic mobile phase consisting of 0.1% aqueous formic acid and acetonitrile (71:29, *v*/*v*), column temperature of 40 °C, and flow rate of 0.35 mL/min (Figure 12) for subsequent investigations.

### 2.5. Qualitative Analysis

Over 13 peaks were detected within 10 min in the mass spectrometry total ion current (TIC) chromatograms obtained in positive and negative modes. The molecular weights of these 13 compounds were determined on the basis of their positive and negative ion mass spectra. 

**Figure 13:**
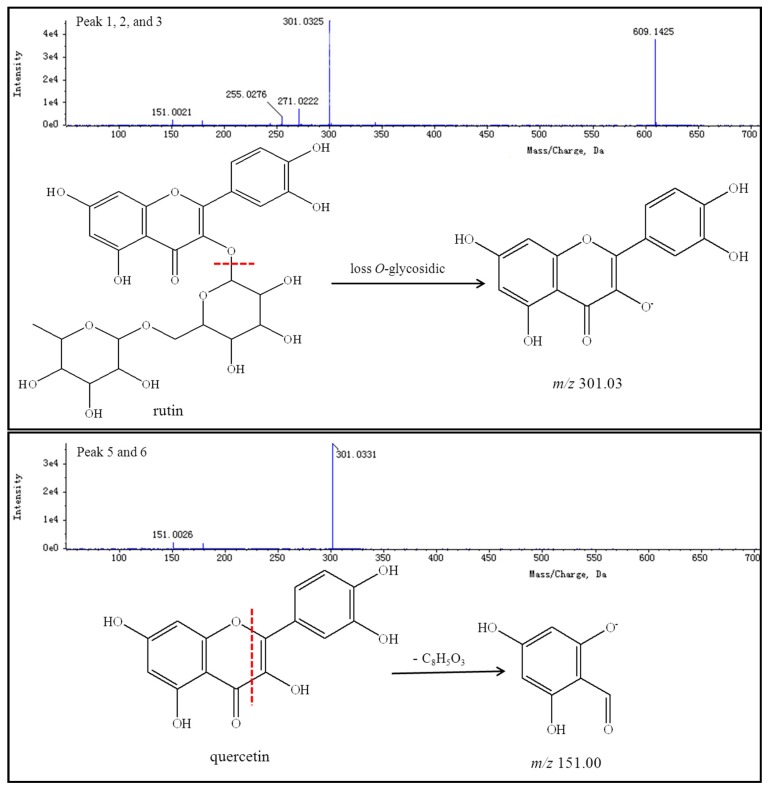

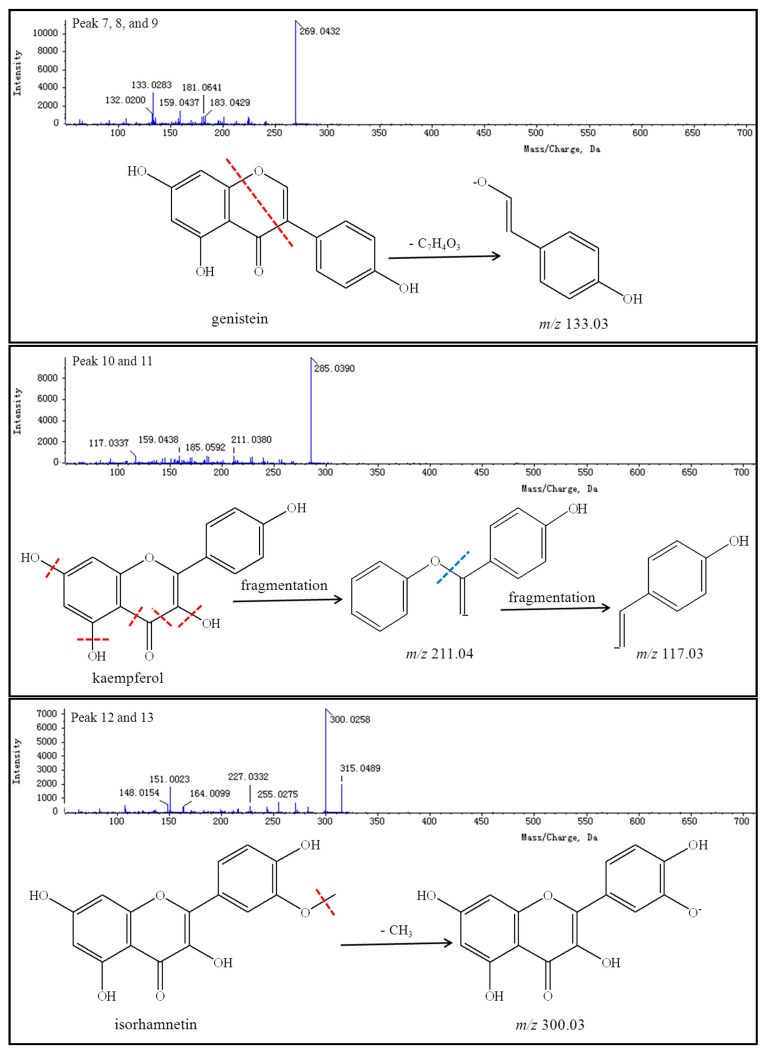
Structure, MS/MS spectra and fragmentation scheme (negative ion mode) of 13 ingredients (the red lines and blue lines are cracking location and ion fragments figure, respectively).

Thirteen peaks, including those of rutin, quercetin, genistein, kaempferol, isorhamnetin, and their isomers, were identified using authentic standards and simultaneously quantified in the FSI extracts. The retention times, formulas, and MS/MS fragmentation data of the 13 compounds are summarized in Table 5. Possible structures and fragmentation schemes are shown in Figure 13; this information was deduced by carefully studying the MS and MS/MS spectral data of each compound and comparing findings with literature values. Peaks 1–3 reveal a group of isomers with a molecular weight of 610 and chemical formula of C_27_H_30_O_16_. The MS^2^ spectra of compounds **1**–**3** showed that the most abundant fragment peak at *m*/*z* 301.03 may be attributed to elimination of *O*-glycosidic groups from the precursor ion at *m*/*z* 609.14. Peaks 1–3 were unambiguously identified as rutin (including its isomers) by comparison with literature values [92,96].

Peaks 5 and 6 indicate a group of isomers with a molecular weight of 302 and chemical formula of C_15_H_10_O_7_. The MS^2^ spectra of compounds **5** and **6** showed the most abundant fragment peaks at *m*/*z* 151.00 (negative ion mode) and *m*/*z* 153.02 (positive ion mode) were appeared. Peaks 5 and 6 were identified as quercetin (including its isomers) by comparison with literature values [83,92,96].

Peaks 7–9 reveal a group of isomers with a molecular weight of 270 and chemical formula of C_15_H_10_O_5_. The MS^2^ spectra of compounds **7**–**9** showed that most the abundant fragment peak at *m*/*z* 133.03 may be attributed to cleavage of C-O and C-C bonds from the precursor ion at *m*/*z* 269.04. Peaks 7–9 were finally identified as genistein (including its isomers) by comparison with literature values [96].

Peaks 10 and 11 reflect a group of isomers with a molecular weight of 286 and chemical formula of C_15_H_10_O_6_. The MS^2^ spectra of compounds **10** and **11** showed the most abundant fragment peaks at *m*/*z* 211.04 and *m*/*z* 117.03 (negative ion mode). Peaks 10 and 11 were subsequently identified as kaempferol (including its isomers) by comparison with literature values [96].

Peaks 12 and 13 demonstrate a group of isomers with a molecular weight of 316 and chemical formula of C_16_H_12_O_7_. The MS^2^ spectra of compounds **12** and **13** showed that the most abundant fragment peak at *m*/*z* 300.03 (negative ion mode) may be attributed to the loss of a methyl radical from the precursor ion at *m*/*z* 315.05. Peaks 12 and 13 were thus identified as isorhamnetin (including its isomers) by comparison with literature values [83,92].

Considering that rutin, quercetin, genistein, kaempferol, and isorhamnetin present extensive biological activities, developing a quality control method based on the content of these compounds is necessary.

### 2.6. Quantitative Analysis

The UHPLC method was fully validated for quantitative determination. Linear regression equations, correlation coefficients, and ranges of calibration curves for the listed compounds are shown in Table 6. Correlation coefficients *R*^2^ > 0.9990 indicated excellent correlations between the concentrations of the investigated compounds and their *PA*s within the tested ranges. Constituent contents were calculated using the relevant calibration curves. The LODs and LOQs of the five compounds were in the range of 13.7661–2684.4763 ng/mL and 24.2013–8948.2543 ng/mL, respectively. Analytical variations in intra- and inter-day (retention time and area) RSD% were less than 2.8330% for all five constituents, as shown in Table 7. The *PA* RSD% of all five constituents after 0, 1, 2, 4, 8, 12, 24, 48, 72, and 96 h was less than 0.4841%; these results imply that the extract solutions are very stable. The RSD% (retention time and area) of six samples from the same batch (repeatability) of material was less than 0.3748% for all five compounds (Table 7). The recoveries of the five compounds exceeded 95.1217%, and RSD% less than 1.8041% (Table 8). These results indicate that the proposed UHPLC method is sensitive, repeatable, and accurate for the quantitative analysis of active compounds in FSI.

## 3. Experimental Section

### 3.1. Chemicals and Materials

Acetonitrile (HPLC-grade), ethanol (HPLC-grade), and methanol (HPLC-grade) were purchased from Fisher Scientific, Inc. (Pittsburgh, PA, USA). Water (HPLC-grade) was purified using a Milli-Q Plus system (Millipore, Bedford, MA, USA). Standard samples of rutin, quercetin, genistein, kaempferol and isorhamnetin were obtained from the National Institutes for Food and Drug Control (Beijing, China). FSI was collected from an experimental field at Beibei District, Chongqing City, and Quanzhou County, Guilin City, Guangxi Autonomous Region in China during harvest time. Materials were confirmed based on morphological, microscopic, and physiochemical analyses according to the Chinese Pharmacopoeia [8]. Voucher specimens were deposited in the Institute of Material Medical Planting, Chongqing Academy of Chinese Materia Medica. Samples were sun-dried and ground into powder.

### 3.2. Experimental Design

#### 3.2.1. Single Factor Tests

The extraction of the five major ingredients under different conditions was investigated by first employing single factor tests to determine the optimal extraction solvent, particle size, extraction frequency, liquid-to-solid ratio, microwave power, and extraction time. The five major ingredients of FSI were extracted using MARS 6 microwave reaction system (CEM Co., Ltd., Matthews, NC, USA). Each extracted solution was passed through a 0.22 μm syringe filter (Tianjin Jinteng Experimental Equipment Co., Ltd., Tianjin City, China) and collected into a 1.5 mL vial. An aliquot (1 μL) of each solution was injected into the UPLC system for analysis.

##### Solvent Selection

Each extract (0.5 g of dried powder sieved through 60 mesh) was mixed with extraction solvent (50 mL of 100% MeOH, 80% MeOH, 60% MeOH, 40% MeOH, 20% MeOH, 100% EtOH, 80% EtOH, 60% EtOH, 40% EtOH or 20% EtOH, pre-leaching 30 min) in a 100 mL-volume tube to determine the optimal extraction solvent. The working microwave power rating was 200 W, and extraction time was 40 s. Experiments were conducted in triplicate (30 experimental treatments), and extraction yield was expressed as a percentage using the following equation: extraction yield (%) = ingredient weight/sample weight × 100.

##### Particle Size Selection

Each extract [0.5 g of dried powder sieved through different meshes (20, 40, 60, 80, and 100 mesh)] was mixed with 100% MeOH (50 mL, based on the results from solvent selection section, pre-leaching 30 min) to determine the optimal particle size. The experiments were conducted in triplicate (15 experimental treatments). Extraction conditions were performed at working microwave power of 200 W and extraction time of 40 s.

##### Extraction Frequency

Each extract (0.5 g of dried powder and sieved through 100 mesh based on the results of particle size selection section) was mixed with 50 mL of 100% MeOH and experiments were conducted 1×, 2×, and 3× to determine the optimal extraction frequency. The working microwave power and time were 200 W and 40 s, respectively. The experiments were also performed in triplicate (9 experimental treatments).

##### Liquid-to-Solid Ratio Selection

Each extract (0.5 g of dried powder) was dissolved in different volumes of 100% MeOH (200, 100, 50, 25, 10 and 5 mL, pre-leaching 30 min) to determine the optimal liquid-to-solid ratio. Experiments were conducted in triplicate (18 experimental treatments) and extracted once (based on the results of extraction frequency section). The working microwave power was 200 W and extraction time was 40 s.

##### Microwave Power Selection

Each extract (0.5 g of dried powder) was mixed with 40% MeOH (25 mL, based on the results of liquid-to-solid ratio selection section, pre-leaching 30 min). Different microwave power levels of 100, 150, 200, 250, 300, and 350 W were applied to determine the best microwave power for extraction. The working time was 40 s. The experiments were performed in triplicate (18 experimental treatments), and extraction was performed once per experiment.

##### Extraction Time Selection

Each extract (0.5 g of dried powder) was mixed with 25 mL of 100% MeOH (pre-leaching 30 min). The working microwave power was 200 W, and extraction was performed once. Extractions were performed for 20, 30, 40, 50, 60, 70, 80, and 90 s to determine the optimal extraction time. Experiments were conducted in triplicate (24 experimental treatments).

##### Central Composite Design

Single-factor tests were employed to determine the preliminary range of the extraction variables, namely, liquid-to-solid ratio (X_1_), microwave power (X_2_), and extraction time (X_3_). A three-factor-five level rotatable central composite design was then employed to determine the combination of extraction variables that would yield the largest amount of five constituents [97,98,99,100]. The coded values of the experimental factors and factor levels were used in the response surface analysis that was run 20× (Table 2) and performed in triplicate. Extraction yields showed that the response variables were fitted to a quadratic polynomial model. The quadratic model for each response was as follows [68]:

(1) $$Y=A_{0}+\sum_{i=1}^{3}A_{i}X_{i}+\sum_{i=1}^{3}A_{ii}X_{i}^{2}+\sum_{i=1}^{2}\sum_{j=i+1}^{3}A_{ij}X_{i}X_{j}$$

where *Y* is the predicted response, *A*_0_ is a constant that fixes the response at the central point of the experiment, *A_i_* is the regression coefficient for the linear effect of term, *X_i_* is the independent variable, *A_ii_* is the quadratic effect term and *A_ij_* is the interaction effect term. The contribution rate was calculated using the following equation [69]:

(2) $$\Delta_{j}=\delta_{j}+\frac{1}{2}\sum_{\begin{array}{l} i=1\\ i\neq j \end{array}}^{m}\delta_{ij}+\delta_{jj},(j=1,2,\cdots ,m);\delta =\left(\begin{array}{cc} 0 & F\leq 1\\ 1-\frac{1}{F} & F\geq 1 \end{array}\right)$$

where Δ*_j_* is the contribution rate, and *F* is the value for the linear effect terms (*F_j_*), interaction effect terms (*F_ij_*), and quadratic effect terms (*F_jj_*, regression coefficients significance test; if using *F* test, direct calculation is performed; if using *t*-test, *F_j_* = *t_j_*^2^, *F_ij_* = *t_ij_*^2^, and *F_jj_* = *t_jj_*^2^, respectively); δ*_j_*, δ*_ij_*, and δ*_jj_* are the calculated *F* values of the independent variables, interaction effect terms, and quadratic effect terms, respectively.

### 3.3. UHPLC Analysis

#### 3.3.1. Preparation of Standard Solution

A mixed standard stock solution of the selected analytes (rutin, 1.9 mg/mL; quercein, 3.8 mg/mL; genistein, 3.1 mg/mL; kaempferol, 3.5 mg/mL; and isorhamnetin, 2.8 mg/mL) was prepared in methanol. Working standard solutions were prepared by diluting mixed standard solutions with methanol to a series of concentrations within the range of 19–380 μg/mL; these solutions were used to plot the calibration curve. Calibration curves were obtained by plotting the peak area (*PA*) *versus* the amount of standards.

#### 3.3.2. Instrumentation and Operating Conditions

UHPLC-ESI-Q-TOF MS/MS analysis was performed on a Shimadzu LC-30AD series UFLC system comprising a diode array detector, vacuum degasser, pump, autosampler, and thermostated column compartment (Shimadzu, Kyoto, Japan) and interfaced to a Triple TOF^®^ 5600 mass analyzer (AB Sciex, Concord, CA, USA) operating in positive and negative ion modes. AB Sciex Analyst software was used to control the LC-MS system and for data acquisition (AB Sciex, Boston, MA, USA). Peakview^®^ 2.2 software (AB Sciex, Boston, MA, USA) was used to process the obtained data.

#### 3.3.3. UHPLC Conditions

Analysis was carried out on a Kinetex C_18_ column (100 mm × 2.1 mm, 2.6 μm, Phenomenex, Torrance, CA, USA). Standards and samples were separated using an isocratic mobile phase consisting of 0.1 % aqueous formic acid and acetonitrile (71:29, *v*/*v*). The detection wavelength was set to 260 nm, and on-line UV spectra were recorded within 200–400 nm. The column temperature was maintained at 40 °C, the flow rate was set to 0.35 mL/min, and the injection volume was 1 μL.

#### 3.3.4. MS Conditions

The following operation parameters were used: ion source gas 1 and ion source gas 2, 55 psi; curtain gas, 25 psi; temperature, 600 °C; ion spray voltage floating, 5500 V; collision energy, 40 V; and collision energy spread, 15 V in positive ion mode (ESI^+^). The parameters in negative mode (ESI^−^) were identical to those in ESI^+^, except for ion spray voltage floating, which was set to 4500 V. For full-scan TOF-MS analysis, a scan range of *m*/*z* 50–1000 with an accumulation time of 150 ms was selected; a scan range of *m*/*z* 50–1000 and accumulation time of 100 ms were used in TOF-MS/MS. MS/MS spectra were acquired in positive and negative ion modes from 0 min to 10 min. Accurate molecular masses of different compounds were obtained by measuring protonated and deprotonated molecules in positive and negative ion modes.

#### 3.3.5. Method Validation for Quantitative Analysis

A calibration curve was generated to confirm the linear relationship between *PA* and the concentrations of the analytes. The limits of detection (LOD) and quantification (LOQ) were defined as the analyte concentrations producing signal-to-noise ratios (S/N) of about 3 and 10, respectively.

*Precision and stability*: The same mixed standard solution was analyzed through six replicates on the same day to assess intra-day variation and once each day for 3 consecutive days to assess inter-day variation. A sample of the medicinal material was prepared as described above and subjected to UHPLC analysis immediately and after 0, 1, 2, 4, 8, 12, 24, 36, 48, 72, and 96 h to detect the *PA* of all five standard compounds for the stability evaluation.

*Repeatability*: Six samples from the same batch (two times, two batches) of medicinal material were extracted and analyzed to evaluate the repeatability of the method.

*Recovery*: Standard constituent mixtures at three concentrations were added to untreated samples. The samples were allowed to settle for 60 min and then extracted and analyzed as described above to determine the recoveries of the five standard compounds. Spiked samples were prepared in triplicate. Recovery was calculated as follows: recovery (%) = (amount found − original amount)/amount spiked × 100.

## 4. Conclusions 

RSM was successfully applied in this study to optimize the microwave-assisted extraction of the five major constituents from FSI. The extraction solvent type, sample particle size, times, liquid-to-solid ratio, microwave power, and extraction time played significant roles in the constituent extraction. Higher yields of the constituents were extracted from FSI using a green extraction solvent, less solvent, and lower extraction power, and simultaneously and considerably decreasing the extraction time. The statistical frequency method indicates that the optimum extraction conditions for the simultaneous extraction of the five components were 100% MeOH, 100 mesh, 50:1, 287 W, and 80 s.

The present study described the development and evaluation of a relatively simple UHPLC and LC-ESI-Q-TOF MS/MS method for the simultaneous analysis of rutin, quercetin, genistein, kaempferol, and isorhamnetin with high sensitivity. The proposed method is simple and rapid, provides high precision, sensitivity, accuracy, and reliability, and is appropriate for detection work. Separation of five compounds of interest was completed within 5 min, and well-resolved peaks were obtained. Run times were significantly shortened. The method developed in this project will be very useful for future studies.

## Figures and Tables

**Table 1:** Extracted conditions of different extraction methods in FSI.

No.	Constituents	Method	Solvent	Ratio	Time (No.)	Time (min)	Temperature (°C)	Extraction Yield (%)
1	Rutin	Unclear	60% ethanol	1:21	1	87	Unclear	13.51
2	Rutin	Untrasound	100% methanol	1:15	1	30	Unclear	0.03~0.04
3	Rutin	Untrasound	100% methanol	1:500	1	30	Unclear	13.88~35.74
4	Rutin	Percolation	100% methanol	1:400	1	40	Room temperature	7.53~10.84
5	Rutin	Untrasound	70% ethanol	1:400	More	40	55	22.27~22.98
6	Rutin	Microwave	55% ethanol	1:20	Unclear	9	Unclear	30.83
7	Rutin	Reflux	100% methanol	1:90	1	420~480	100	12.77
8	Rutin	Microwave	Water	1:30	3	4	Unclear	12.63
9	Rutin	Untrasound	70% ethanol	1:30	3	30	Unclear	12.81
10	Rutin	Untrasound	60% ethanol	1:10	Unclear	20	45	6.1
11	Rutin	Soxhlet	100% methanol	1:250	Unclear	Unclear	100	19.25 and 26.27
12	Rutin	Percolation	80% ethanol	Unclear	Unclear	Unclear	45	11.6
13	Rutin	Untrasound	Alkaline solution	1:15	2	10	20	Unclear
14	Rutin	Untrasound	Alkaline solution	1:20	2	30	60	18.25
15	Rutin	Microwave	Water	1:100	1	16	Unclear	21.97
16	Rutin	Microwave	100% methanol	Unclear	1	Unclear	Unclear	Unclear
17	Rutin	Microwave	Water	Unclear	4	24	Unclear	17
18	Rutin	Microwave	100% ethanol	1:20	1	9	Unclear	Unclear
19	Rutin	Microwave	65% ethanol	1:18	1	4	Unclear	Unclear
20	Rutin	Microwave	Water	1:100	3	10	Unclear	14.66
21	Rutin	Enzymatic	0.03% cellulase	1:55	1	120	45	19.82
22	Rutin	Untrasound	Alkaline solution	1:15	2	10	Room temperature	19.16
23	Rutin	Basic	Alkaline solution	1:25	1	720	Room temperature	11
24	Rutin	Unclear	100% methanol	1:30	1	210	90	Unclear
25	Rutin	Supercritical fluid CO_2_	Ether	Unclear	Unclear	420	50	6.88
26	Rutin	Untrasound	Water	1:20	2	20	70	Unclear
27	Rutin	Untrasound	100% methanol	1:500	1	30	Unclear	≥15%
28	Rutin	infrared-assisted	70% methanol	1:30	1	4.8	Unclear	25.26
29	Quercetin	Soxhlet and Reflux	Ether and methanol	1:20	3	60	100	0.87
30	Quercetin	Microwave	75% ethanol	1:8	1	10	Unclear	0.46~0.61
31	Quercetin	Reflux	80% ethanol	1:12.5	1	120	100	10.01~10.40
32	Quercetin	Microwave	100% ethanol	1:4	3	10	Unclear	0.57
33	Quercetin	Basic	Alkaline solution	1:35	1	1440	Unclear	Unclear
34	Quercetin	Decoction	0.05% NaOH	1:10	4	20	100	Unclear
35	Flavonoid	Untrasound	60% ethanol	1:15	2	30	75	8.46
36	Flavonoid	Untrasound	99.8% borax	1:10	2	30	35	18.8
37	Flavonoid	Untrasound	60% ethanol	1:27	2	60	60	Unclear
38	Flavonoid	Reflux	40% ethanol	1:2	3	120	100	13.04
39	Flavonoid	High-Pressure	50% ethanol	1:40	1	20	130	19.4
40	Flavonoid	Microwave	70% ethanol	1:10	1	30	Unclear	Unclear
41	Flavonoid	Untrasound	70% ethanol	1:50	1	40	Unclear	46.31
42	Flavonoid	Untrasound	60% ethanol	1:8	3	60	80	15.03
43	Rutin Quercetin	Untrasound	100% methanol	1:500	1	30	Unclear	24.42~24.51; 1.39~1.43
44	Rutin Quercetin	Untrasound	60% ethanol	Unclear	1	Unclear	Room temperature	Unclear
45	Rutin Quercetin	Soxhlet	100% methanol	1:10	1	Unclear	100	13.88; 0.15; 10.48; 0.22
46	Rutin Quercetin Genistein	Untrasound	100% ethanol	Unclear	Unclear	20	Unclear	Unclear

**Table 2:** Extraction yield of response surface CCD (*n* = 3) and expressed as means ± SD (units: %).

No.	X_1_	X_2_ (W)	X_3_ (S)	Rutin	Quercetin	Genistein	Kaempferol	Isorhamnetin
1	−1 (50)	−1 (200)	−1 (60)	16.85 ± 0.28	2.04 ± 0.30	0.002 ± 0.00	0.059 ± 0.00	0.11 ± 0.01
2	−1 (50)	−1 (200)	1 (80)	21.05 ± 0.33	5.29 ± 0.16	0.002 ± 0.00	0.059 ± 0.00	0.15 ± 0.00
3	−1 (50)	1 (300)	−1 (60)	20.70 ± 1.20	4.78 ± 0.078	0.002 ± 0.00	0.044 ± 0.00	0.13 ± 0.01
4	−1 (50)	1 (300)	1 (80)	33.76 ± 0.44	6.19 ± 0.09	0.003 ± 0.00	0.065 ± 0.00	0.18 ± 0.00
5	1 (100)	−1 (200)	−1 (60)	16.00 ± 0.02	1.62 ± 0.12	0.002 ± 0.00	0.058 ± 0.00	0.12 ± 0.01
6	1 (100)	−1 (200)	1 (80)	18.73 ± 0.38	4.46 ± 0.22	0.001 ± 0.00	0.058 ± 0.01	0.14 ± 0.01
7	1 (100)	1 (300)	−1 (60)	18.81 ± 0.12	2.67 ± 0.10	0.001 ± 0.00	0.057 ± 0.00	0.13 ± 0.00
8	1 (100)	1 (300)	1 (80)	23.25 ± 0.33	3.48 ± 0.21	0.001 ± 0.00	0.056 ± 0.00	0.15 ± 0.01
9	−1.682 (32.95)	0 (250)	0 (70)	23.24 ± 0.07	4.39 ± 0.19	0.002 ± 0.00	0.063 ± 0.00	0.16 ± 0.00
10	1.682 (117.05)	0 (250)	0 (70)	18.93 ± 0.35	2.48 ± 0.19	0.001 ± 0.00	0.059 ± 0.00	0.13 ± 0.01
11	0 (75)	−1.682 (165.9)	0 (70)	17.45 ± 0.28	2.71 ± 0.19	0.002 ± 0.00	0.057 ± 0.00	0.11 ± 0.01
12	0 (75)	1.682 (334.1)	0 (70)	24.99 ± 0.43	4.80 ± 0.07	0.002 ± 0.00	0.056 ± 0.00	0.16 ± 0.00
13	0 (75)	0 (250)	−1.682 (53.18)	12.54 ± 0.12	2.13 ± 0.26	0.001 ± 0.00	0.050 ± 0.00	0.11 ± 0.00
14	0 (75)	0 (250)	1.682 (86.82)	24.50 ± 0.45	4.88 ± 0.11	0.002 ± 0.00	0.058 ± 0.00	0.15 ± 0.00
15	0 (75)	0 (250)	0 (70)	29.10 ± 0.20	5.42 ± 0.36	0.002 ± 0.00	0.065 ± 0.00	0.17 ± 0.01
16	0 (75)	0 (250)	0 (70)	29.14 ± 0.40	5.34 ± 0.12	0.002 ± 0.00	0.063 ± 0.00	0.16 ± 0.01
17	0 (75)	0 (250)	0 (70)	27.29 ± 0.34	5.28 ± 0.08	0.002 ± 0.00	0.063 ± 0.00	0.16 ± 0.00
18	0 (75)	0 (250)	0 (70)	27.15 ± 0.07	5.26 ± 0.14	0.002 ± 0.00	0.062 ± 0.00	0.16 ± 0.01
19	0 (75)	0 (250)	0 (70)	28.29 ± 0.05	5.08 ± 0.03	0.002 ± 0.00	0.061 ± 0.00	0.16 ± 0.00
20	0 (75)	0 (250)	0 (70)	27.08 ± 0.12	5.72 ± 0.18	0.003 ± 0.00	0.068 ± 0.00	0.17 ± 0.01

**Table 3:** Regression coefficients of predicted polynomial models for the investigated responses from FSI extracts.

Coefficient	Constituents				
	Rutin	Quercetin	Genistein	Kaempferol	Isorhamnetin
A_0_	27.9635 ***	5.3376 ***	0.0024 ***	0.0638	0.1622 ***
A_1_	−1.6697 **	−0.6794 ***	−3.164 × 10^−4^ ***	−3.298 × 10^−4^	−0.0056 **
A_2_	26783 ***	0.5296 ***	2.961 × 10^−5^	−9.444 × 10^−4^	0.0113 ***
A_3_	3.2617 ***	0.9475 ***	1.222 × 10^−4^ *	0.0024 *	0.0143 ***
A_11_	−2.1643	−0.6022	−1.529 × 10^−4^	−9.858 × 10^−4^	−0.0061
A_22_	−2.1159	−0.4883	−2.569 × 10^−4^	−0.0024	−0.0092
A_33_	−3.0719	−0.5757	−2.648 × 10^−4^	−0.0036	−0.0101
A_12_	−1.1534 *	−0.4454 **	−1.505 × 10^−4^ *	6.392 × 10^−4^	−0.0056 *
A_13_	−1.2602 *	−0.1257	−1.938 × 10^−4^ *	−0.0028 *	−0.0056 *
A_23_	1.3205 *	−0.4827 **	1.619 × 10^−4^ *	0.0026 *	0.001
Model	***	***	***	***	***
Lack of fit	ns	ns	ns	ns	ns
R^2^	0.963	0.974	0.933	0.828	0.957
R^2^_adj_	0.931	0.95	0.872	0.673	0.918

ns, Not significant at *p* ≤ 0.05; * Significant at *p* ≤ 0.05; ** Significant at *p* ≤ 0.01; *** Significant at *p* ≤ 0.0001.

**Table 4:** Estimated optimum conditions, predicted and experimental values of responses under these conditions.

Response Variables	Optimum Extraction Conditions (Obtained from Equation)			Maximum Extraction Yields (%)		Extraction Yields at Optimal Conditions from the Statistical Frequency Method (%)
	Liquid-to-Solid Ratio	Microwave Power (W)	Time (s)	Predicted	Actual	
Rutin	50 (50)	310 (311)	80 (80)	32.06	32.17	32.11 ^ns^
Quercetin	54 (53.5)	281 (280.5)	77 (76.6)	6.1	6.23	6.26 ^ns^
Genistein	33 (33)	295 (295)	81 (81.2)	0.003	0.003	0.002 **
Kaempferol	36 (36.3)	258(257.5)	80 (80.1)	0.06	0.06	0.06 ^ns^
Isorhamnetin	40 (39.8)	305 (305)	81(81.2)	0.18	0.18	0.17^ns^

** Compared with the significant for actual yield of optimum conditions and statistical frequency conditions at *p* ≤ 0.01; ^ns^, Not significant at *p* ≤ 0.01 or *p* ≤ 0.05.

**Table 5:** Precursor and product ion of the ingredients in the methanol extract of FSI in UHPLC-ESI-Q-TOF MS/MS.

No.	RT (min)	Name	Formula	[M + H]^+^[M − H]^−^	Expected *m/z*	Experimental *m/z*	Fragments	Error (ppm)
1	0.759	rutin	C_27_H_30_O_16_	[M − H]^−^	609.1398	609.1404	301.0325, 270.0222, 255.0275	1.00
2	0.810	rutin	C_27_H_30_O_16_	[M − H]^−^	609.1401	609.1406	301.0325	0.70
3	0.855	rutin	C_27_H_30_O_16_	[M − H]^−^	609.1379	609.1391	301.0310, 270.0215, 255.0271	1.90
4	1.176	unknown	-	[M − H]^−^	577.1495	577.1497	-	0.40
				[M + H]^+^	579.1791	579.1790	147.0433, 119.0481	−0.10
5	2.159	quercetin	C_15_H_10_O_7_	[M − H]^−^	301.0317	301.0316	151.0029	−0.10
				[M + H]^+^	303.0519	303.0521	229.0505, 153.1069	0.70
6	2.256	quercetin	C_15_H_10_O_7_	[M − H]^−^	301.0309	301.0315	151.0025	1.90
				[M + H]^+^	303.0515	303.0519	229.0501, 153.0174	1.40
7	3.447	genistein	C_15_H_10_O_5_	[M − H]^−^	269.0421	269.0422	133.0278	0.30
				[M + H]^+^	271.0618	271.0619	-	0.40
8	3.510	genistein	C_15_H_10_O_5_	[M − H]^−^	269.0429	269.0623	133.0283	−2.30
				[M + H]^+^	271.0613	217.0616	-	0.80
9	3.614	genistein	C_15_H_10_O_5_	[M − H]^−^	269.0340	269.0413	133.0278	1.40
10	3.886	kaempferol	C_15_H_10_O_6_	[M − H]^−^	285.0369	285.0367	211.0380, 117.0338	−0.60
				[M + H]^+^	287.0569	287.0568	-	−0.30
11	4.038	kaempferol	C_15_H_10_O_6_	[M − H]^−^	285.0359	285.0363	-	1.40
12	4.267	isorhamnetin	C_16_H_12_O_7_	[M − H]^−^	315.0465	315.0464	300.0254, 151.0023	−0.40
				[M + H]^+^	317.0680	317.0679	-	−0.40
13	4.313	isorhamnetin	C_16_H_12_O_7_	[M − H]^−^	315.0463	315.0464	300.0237, 151.0016	0.40

**Table 6:** Linear regression data, LOD, and LOQ of the investigated compounds.

Analytes	Linear Regression Data			LOD (ng/mL)	LOQ (ng/mL)
	Regression Equations	Linear Ranges (μg/mL)	*R* ^2^		
Rutin	y = 5114520.5x + 94252.745	1.9–950	0.9994	26.8447	89.4825
Quercetin	y = 12527006x - 107866.6	3.8–1900	0.9999	40.2142	96.3997
Genistein	y = 16908433x + 523731.7	3.1–1550	0.9990	41.7210	98.3375
Kaempferol	y = 12102061x − 18222.25	3.5–1750	1.0000	13.7661	24.2013
Isorhamnetin	y = 12274446x − 30971.9	2.8–1400	1.0000	15.7581	28.7221

**Table 7:** Intra- and inter-day precision of constituents; results are shown as RSD % (*n* = 6).

Analytes	Intra-Day Variability		Inter-Day Variability						Repeatability	
	Retention Time	Are	Day 1 Time	Day 1 Area	Day 2 Time	Day 2 Area	Day 3 Time	Day 3 Area	Time	Area
Rutin	0.3085	0.6852	0.0128	0.0042	0.0049	0.0157	0.0056	0.0101	0.0027	0.2095
Quercetin	0.0994	0.4892	0.0275	0.0083	0.0151	0.2064	0.0126	0.6322	0.0010	0.2085
Genistein	0.1536	0.0526	0.0298	0.2502	0.0107	0.6464	0.0130	0.0179	0.0006	0.3748
Kaempferol	0.1324	2.8330	0.0242	0.0320	0.0130	0.3355	0.0106	0.0233	0.0007	0.2086
Isorhamnetin	0.0997	0.9402	0.0253	0.0049	0.0093	0.0058	0.0078	0.0193	0.0009	0.2430

**Table 8:** Recovery studies of rutin (**1**), quercetin (**2**), genistein (**3**), kaempferol (**4**), and isorhamnetin (**5**).

Series No.	Compound	Theoretical (mg)	Found (mg)	Recovery (%)
1	**1**	40.1761	40.1724 ± 0.1217	96.1951 ± 0.3029
	**2**	0.3280	0.3276 ± 0.0021	99.8091 ± 0.6117
	**3**	0.1559	0.1583 ± 0.0028	101.5719 ± 1.8041
	**4**	0.1897	0.1829 ± 0.0021	96.1354 ± 1.1425
	**5**	0.1632	0.1636 ± 0.0018	100.2918 ± 1.1584
2	**1**	40.2711	40.2654 ± 0.1496	97.0428 ± 0.3715
	**2**	0.5180	0.5181 ± 0.0012	100.0234 ± 0.24505
	**3**	0.3109	0.3158 ± 0.0027	101.6114 ± 0.8989
	**4**	0.3647	0.3476 ± 0.0023	95.1217 ± 0.6555
	**5**	0.3032	0.2958 ± 0.0013	97.3578 ± 0.4417
3	**1**	40.4611	40.4707 ± 0.2773	102.5346 ± 0.6855
	**2**	0.8980	0.9018 ± 0.0099	100.5011 ± 1.1056
	**3**	0.6209	0.6208 ± 0.0023	99.9971 ± 0.3787
	**4**	0.7147	0.6923 ± 0.0039	96.8074 ± 0.5489
	**5**	0.5832	0.5711 ± 0.0021	97.8323 ± 0.3439
Average	**1**			98.5908636
	**2**			100.1112057
	**3**			101.0601425
	**4**			96.02157394
	**5**			98.49388817
